# Paracetamol and ibuprofen combination for the management of acute mild-to-moderate pain in children: expert consensus using the Nominal Group Technique (NGT)

**DOI:** 10.1186/s13052-023-01445-4

**Published:** 2023-03-21

**Authors:** Niccolò Parri, Davide Silvagni, Alberto Chiarugi, Elisabetta Cortis, Antonio D’Avino, Marcello Lanari, Paola Giovanna Marchisio, Cesare Vezzoli, Stefania Zampogna, Annamaria Staiano

**Affiliations:** 1 Department of Emergency Medicine and Trauma Center, Meyer Children’s Hospital IRCCS, Florence, Italy; 2 Pediatric Emergency Unit, Department of Neonatal and Pediatric Critical Care, University Hospital of Verona, Verona, Italy; 3 Section of Clinical Pharmacology and Oncology, Department of Health Sciences, University of Florence, Florence, Italy; 4 Headache Center and Clinical Pharmacology Unit, Careggi University Hospital, Florence, Italy; 5 UOC of Pediatrics, Sant’Eugenio Hospital, Rome, Italy; 6 President of FIMP (Italian Federation of Primary Care Pediatricians), Naples, Italy; 7 Pediatric Emergency Unit, IRCCS Azienda Ospedaliera Universitaria Di Bologna, Bologna, Italy; 8 Fondazione IRCCS Ca’ Granda Ospedale Maggiore Policlinico, Milan, Italy; 9 University of Milan, Milan, Italy; 10 Pediatric Intensive Care Unit, Children’s Hospital, ASST-Spedali Civili Brescia, Brescia, Italy; 11 Department Pediatrics, Azienda Sanitaria Di Crotone President of SIMEUP (Italian Society of Pediatric Emergency Medicine Urgency), Crotone, Italy; 12 Department of Translational Medical Sciences, Section of Pediatrics, University of Naples “Federico II”, President of SIP (Italian Society of Pediatric), Naples, Italy

**Keywords:** Ibuprofen, Paracetamol, Fixed-dose, Pediatric, Children, Acute pain

## Abstract

**Background:**

Acute pain is a common symptom in children of all ages, and is associated with a variety of conditions. Despite the availability of guidelines, pain often remains underestimated and undertreated. Paracetamol and ibuprofen are the most commonly used drugs for analgesia in Pediatrics. Multimodal pain management by using a combination of paracetamol and ibuprofen results in greater analgesia.

**Methods:**

An investigation using the Nominal Group Technique was carried out between May and August 2022. Two open (non-anonymous) questionnaires were consecutively sent to a Board of ten clinicians to understand their opinions on the use of the oral paracetamol and ibuprofen association. Answers were examined in a final meeting where conclusions were drawn.

**Results:**

The board achieved a final consensus on a better analgesic power of paracetamol and ibuprofen in fixed-dose combination as compared to monotherapy, without compromising safety. Strong consensus was reached on the opinion that the fixed-dose combination of paracetamol and ibuprofen may be a useful option in case of inefficacy of one or other drug as monotherapy, especially in case of headaches, odontalgia, earache, and musculoskeletal pain. The use of the fixed combination may be also considered suitable for postoperative pain management.

**Conclusions:**

The use of the fixed-dose combination may represent advantage in terms of efficacy and safety, allowing a better control of the dose of both paracetamol and ibuprofen as monotherapy, thus minimizing the risk of incorrect dosage. However, the limited evidence available highlights the need for future well designed studies to better define the advantages of this formulation in the various therapeutic areas.

**Supplementary Information:**

The online version contains supplementary material available at 10.1186/s13052-023-01445-4.

## Background

Acute pain is a common symptom in pediatric age. The underlying cause of pain can be medical (e.g., headache, earache, trauma) or surgical (e.g. postoperative pain) [1]. Regardless of its origin, pain can weaken the child’s physical and psychological integrity, and ultimately be a cause of stress for parents. If left untreated, pain may impact the quality of life and determine short- and long-term effects, with patients becoming more sensitive and getting more pain with less provocation in their lifetime [2].

Therefore, pain should always be evaluated with age-appropriate scales and treated adequately in each patient [3].

Despite the availability of guidelines [1, 4], pain in children is often underestimated, and as a consequence undertreated [4, 5] both in patients seen in emergency departments and in patients admitted to hospitals [3].

According to the International Association for the Study of Pain (IASP), multimodal analgesia is one of the possible approaches to manage acute pain [1].

Multimodal analgesia is a pharmacologic method of pain management which combines various groups of medication for pain relief with fewer side effects than single analgesics [1].

Ibuprofen and paracetamol which are commonly used as first-line treatment for acute pain with the same level of evidence, and a comparable efficacy and safety profile, are both recommended for multimodal analgesia [2].

The aim of this research was to share experts’ opinions about the most appropriate use of oral paracetamol and ibuprofen in fixed-dose combination (3.3:1 dose ratio) for the treatment of acute mild-to-moderate pain in children.

## Methods

We conducted a Nominal Group Technique (NGT) investigation on the use of oral paracetamol and ibuprofen fixed-dose combination for the treatment of acute mild-to-moderate pain in children.

**Fig. 1:**
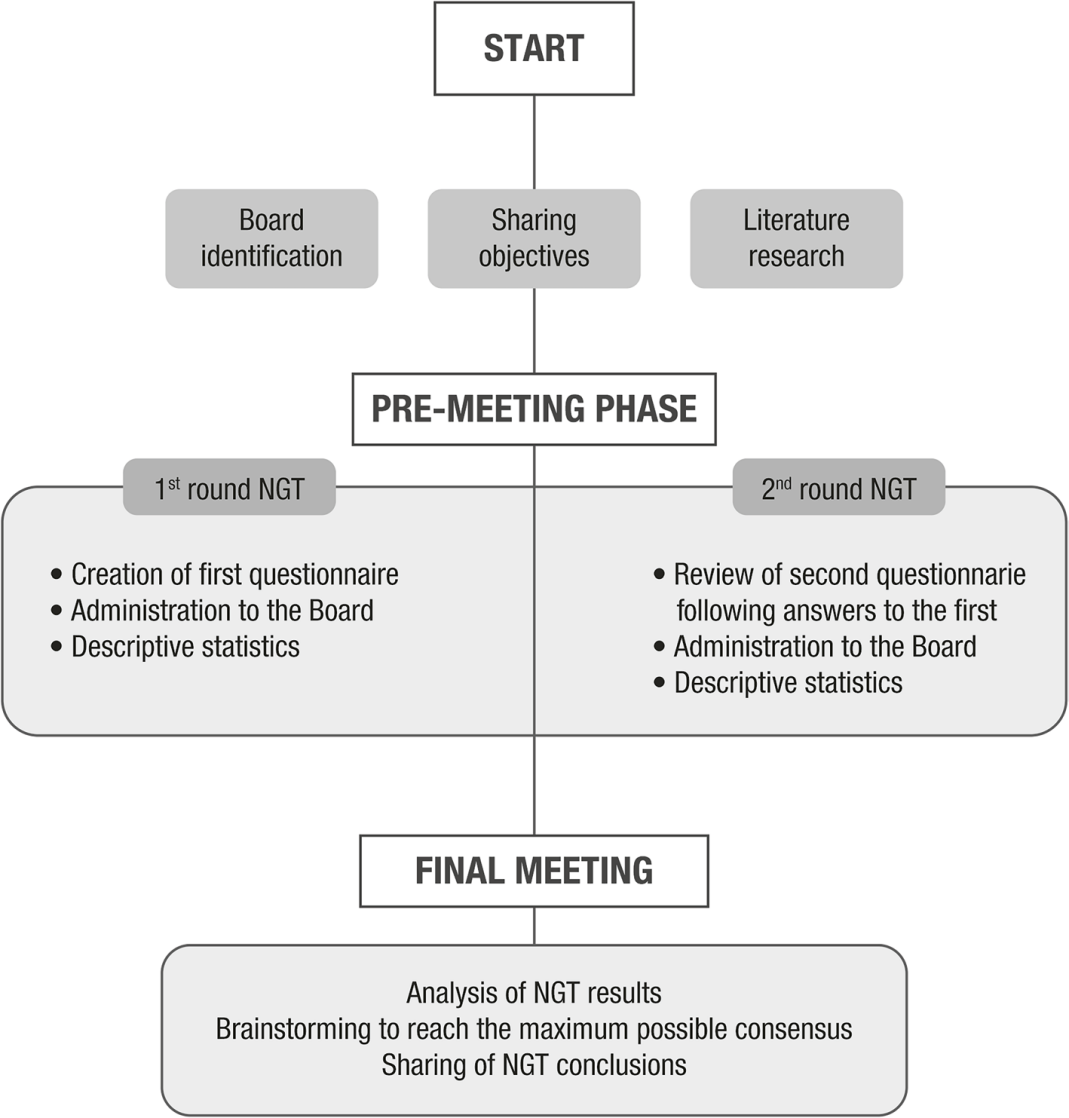
Scheme of modified NGT method

The NGT is a suitable method for generating ideas and consensus in a virtual format [6]. It represents a direct and structured technique based on experts’ opinion. This study used a modified NGT which included three different phases: initial phase, pre-meeting phase, final meeting phase (Fig. 1).

### Initial phase

The initial phase included the Board identification and the literature search.

### Board identification and sharing of objectives

A medical content factory identified a Board composed of nine clinicians with experience in pediatric pain management and one pharmacologist based on their institutional affiliations and if satisfying two of the following selection criteria: documented clinical experience in one of the branches of pediatrics, being active member of scientific societies dealing with pediatric patients and pain management, publications with pediatric pain as topic (at least five in the last five years), and different background (e.g., general practitioner vs hospitalist, size of the hospital, geographic location in the country).

The survey objectives were identified by reviewing the most recent publications [7, 8] and guidelines [1, 4] on acute pain. Objectives were shared with the Board in order to collect valuable opinions and suggestions for the next step.

### Literature research

We performed a five-year (April 2017-April 2022) literature search within the PubMed database to identify the topics of the survey. The search was focused on the following keywords: paracetamol, ibuprofen, children, postoperative, headache, rheumatic pain, fixed dose, earache, musculoskeletal pain.

Filters applied during the PubMed search: in the last 5 years, Preschool Child: 2–5 years, Child: 6–12 years; Observational Study (prospective cohorts or retrospective cohorts), Review, Meta-Analysis, Systematic Reviews, Clinical Trial, Classical Article, Humans, English.

The search terms are reported in Additional file 1.

Duplicates were removed from the list of publications. Articles were screened by title and abstract to check the appropriateness of the contents with respect to the research objective. References of the selected articles were screened to search for other possibly missing articles, left out by our research.

Relevant publications on the effecacy, pharmacokinetics, and safety of the paracetamol/ibuprofen fixed-dosecombination for the treatment of acute pain [7–9] were analysed. National and international guidelines for the management of pain in children [1, 4] were also reviewed. The analysis also focused on even more critical aspects related to the use of ibuprofen and paracetamol in clinical practice: pain undertreatment, appropriateness of dosage regimen, compliance with guidelines by healthcare professionals, and parents [10, 11].

### Pre-meeting phase and questionnaires

Based on the results of the literature research, a first semi-structured open (non-anonymous) survey consisting of ten questions was developed (Table 1). The questionnaire was emailed to the Board members on May 2022 with a copy of the bibliographic search.

**Table 1:** First questionnaire^a^ administered to the Board

Question No	Question	Answers
1	Would you use the fixed combination for the treatment of postoperative pain?	Yes/No If so, in which cases? If not, why? (Open field)
2	The fixed combination in the pediatric setting is effective in case of:	Select one or more options: Headaches Rheumatic pain Earache Post-traumatic musculoskeletal pain Odontalgia Other (open field)
3	The fixed combination in the pediatric setting is ineffective in case of:	Select one or more options: Headaches Rheumatic pain Earache Post-traumatic musculoskeletal pain Odontalgia Other (open field)
4	In the pediatric field, the oral suspension of the fixed combination would be preferred because:	Select one or more options: It allows a simple and accurate definition of the dose as a function of body weight It guarantees a homogeneous trend with the variation of the weight of the child and uniformity in the frequency of administration It allows to minimize the variability between dosages in terms of mg/kg of the active ingredients administered It guarantees greater regularity and completeness of the absorption of the active ingredient Other (open field)
5	What are the factors that could make the fixed combination effective?	Select one or more options: Complementarity of the mechanisms of action Synergy between the effects of the two substances Reduction of the dosage of ibuprofen Reduction of the maximum daily dose of each drug Increased time interval between drug administration Reduction of the risk of incorrect administration of individual active ingredients Other (open field)
6	What do you think are the possible implications in terms of safety and tolerability of the fixed combination?	Select one or more options: Safety especially in patients with comorbidities or in polytherapy Superior analgesia compared to single drugs used alone without compromising tolerability Increased risk of gastrointestinal side effects Increased risk of hepatic side effects Other (open field)
7	What do you think is the pharmacodynamic advantage of the fixed combination?	Select one of the options: Greater analgesic power Greater anti-inflammatory power Both of them Neither
8	In case of ineffectiveness of paracetamol alone, would you use the fixed combination?	Yes/No If so, in which cases? If not, why? (Open field)
9	In case of ineffectiveness of ibuprofen alone, would you use the fixed combination?	Yes/No If so, in which cases? If not, why? (Open field)
10	Would you use the combination for the treatment of discomfort (defined as mild or moderate acute pain)?	Yes/No If so, in which cases? If not, why? (Open field)

^a^In this questionnaire, "fixed combination" means the simultaneous administration of paracetamol and ibuprofen at a fixed dose in a 3.3:1 ratio

Once collected, the results of the first questionnaire were analysed. Questions were validated if there was an agreement of ≥ 7members. On the other hand, questions were not validated if ≤ 3 members reached the agreement.

The questions and/or options that received an agreement of 4 to 6 Board members were reviewed, amended, and proposed again in a second questionnaire of the NGT survey, after an evaluation phase that involved the Board members and/or the acquisition of supporting literature. All the answers to the open questions provided by the Board were raised as multiple-choice questions.

The second questionnaire included a new multiple-choice question on the willingness of board members to use the fixed-dose combination of paracetamol and ibuprofen for the treatment of acute pain (Table 2). The second open (non-anonymous) survey was administered to the Board, starting on July 2022. Once the responses to the second questionnaire were acquired, they were processed in the form of a descriptive statistical analysis and the materials for the final meeting with the Board were prepared. The final remote meeting, on 31 August 2022 was conducted by a facilitator. All Board members were present at the meeting.

**Table 2:** Second questionnaire^a^ administered to the Board

Question No	Question	Answers
1	In which cases would you use the fixed combination for the treatment of postoperative pain (having assessed the antiplatelet effect of ibuprofen in order not to compromise hemostasis)?	Select one or more options: ENT surgery Eye surgery Dental surgery Skin and soft tissue surgery Odontalgia Orthopedic surgery Abdominal surgery When paracetamol alone does not control pain When both anti-inflammatory and analgesic effects are desired Never
2	The fixed combination in the pediatric setting is effective in case of:	Select one or more options: Acute rheumatic pain Chronic rheumatic pain Abdominal pain
3	In the pediatric field, the oral suspension of the fixed combination would be preferred because:	Select one or more options: It maximizes the correctness of the dosage in relation to the weight of the child It promotes regularity and completeness of the absorption of the active ingredients It overcomes the child's frequent aversion to other pharmaceutical forms (tablets, suppositories) It allows for quick pain control with greater effectiveness It reduces parental dosing errors compared to the combined administration of two separate drugs None of the above
4	The combination implies greater safety in patients suffering from concomitant diseases and/or undergoing additional treatments	Select one of the options: Yes/No
5	What do you think is the pharmacodynamic advantage of the fixed combination?	Select one of the options: Greater analgesic power Greater anti-inflammatory power Both of them
6	In case of ineffectiveness of paracetamol alone, would you use the fixed combination?	Select one or more options: Headaches Acute rheumatic pain Chronic Rheumatic Pain Earache Post-traumatic musculoskeletal pain Odontalgia Abdominal pain In cases selected exclusively based on clinical judgment (physician's preferences) Moderate pain that does not respond to the administration of paracetamol alone in the first instance Never
7	In case of ineffectiveness of ibuprofen alone, would you use the fixed combination?	Select one or more options: Headaches Acute rheumatic pain Chronic Rheumatic Pain Earache Post-traumatic musculoskeletal pain Odontalgia Abdominal pain In cases selected exclusively based on clinical judgment (physician's preferences) Moderate pain that does not respond to the administration of paracetamol alone in the first instance Never
8	Would you use the fixed combination for acute pain treatment? ^b^	Select one or more options: Mild (score 1–3) Moderate (score 4–6) Severe (score 7–10) None

^a^In this questionnaire, "fixed combination" means the simultaneous administration of paracetamol and ibuprofen at a fixed dose in a 3.3:1 ratio

^b^Reference was made to the scales approved and used in Italy depending on age: FLACC (Face-Legs-Activity-Crying-Consolability) for children < 3 years, WONG-BAKER for children up to 8 years, NRS (Numeric Rating Scale) for older children

The meeting started with a summary of the results obtained from the NGT survey, followed by a brainstorming session. The debate concerned both the already validated questions and those which did not reach a qualified majority. In the final meeting the results of the two questionnaires were shared to confirm the questions validated or to reach the highest possible consensus for those with intermediate score (i.e. agreement 4–6 members). The aim of the meeting was to share experts’ opinions about the most appropriate use of oral paracetamol and ibuprofen in fixed-dose combination for pain management in children.

## Results

At the end of this NGT survey, our Board has reached a consensus on 12 questions (Table 3). Among these, questions n. 1, 3, 5, 6, 7, 8, 10 and 12 have been approved in the second round (agreement ≥ 7) and confirmed at the final meeting; the questions n. 1, 5, 8 and 10 had already been validated at the first round. Instead, the questions n. 2, 4, 9 and 11 have been discussed by the Board during the final meeting, because some of their response options had not reached a qualified majority at the second questionnaire.

**Table 3:** Questions approved at final meeting and clinician' responses

**N**	**Question**	**Option answers validated at the second questionnair**	**Clinician’ answers (*n* = 10)**	**Approved at final meeting**
1	Would you use the fixed combination for the treatment of postoperative pain?	Option Yes (*validated at the first round)*	9	*Confirmed consensus at the final meeting*
2	Derived from the open field of the first round In which cases would you use the fixed combination for the treatment of postoperative pain (having assessed the antiplatelet effect of ibuprofen in order not to compromise hemostasis)?	When both anti-inflammatory and analgesic effects are desired* (validated at the first round)*	8	*Confirmed consensus at the final meeting*
		When paracetamol alone does not control pain	6	*Agreed consensus following brainstorming*
3	The fixed combination in the pediatric setting is effective in case of:	Odontalgia *(validated at the first round)*	10	*Confirmed consensus at the final meeting*
		Headaches *(validated at the first round)*	9	*Confirmed consensus at the final meeting*
		Earache *(validated at the first round)*	8	*Confirmed consensus at the final meeting*
		Musculoskeletal pain *(validated at the first round)*	8	*Confirmed consensus at the final meeting*
		Chronic rheumatic pain (*option derived from the open field of first round*)	8	*Confirmed consensus at the final meeting*
4	Reintroduced with rewording of the answer options based on first round results In the pediatric field, the oral suspension of the fixed combination would be preferred because:	It optimizes the dosage in relation to the weight of the child *(reworded from the first round)*	7	*Confirmed consensus at the final meeting*
		It reduces parental dosing errors compared to the combined administration of two separate drugs *(derived from the open field of first round*)	9	*Confirmed consensus at the final meeting*
		It allows for quick pain control with greater effectiveness *(derived from the open field of first round)*	5	*Agreed consensus* *following brainstorming*
5	What are the factors that could make the fixed combination effective?	Complementarity of the mechanisms of action *(validated at the first round)*	9	*Confirmed consensus at the final meeting*
		Synergy between the effects of the two substances *(validated at the first round)*	7	*Confirmed consensus at the final meeting*
6	What do you think are the possible implications in terms of safety and tolerability of the fixed combination?	Superior analgesia compared to single drugs used alone without compromising tolerability *(validated at the first round)*	9	*Confirmed consensus at the final meeting*
7	Reintroduced for intermediate agreement of the first round What do you think is the pharmacodynamic advantage of the fixed combination?	Greater analgesic power	8	*Confirmed consensus at the final meeting*
8	In case of inefficacy of paracetamol alone, would you use the fixed combination?	Option Yes *(validated at the first round)*	8	*Confirmed consensus at the final meeting*
9	Derived from the open field of first round In case of inefficacy of paracetamol alone, would you use the fixed combination?	Headaches	5	*Agreed consensus to all options at the last meeting*^a^
		Earache	6	
		Post-traumatic musculoskeletal pain	9	
		Odontalgia	7	
		Moderate pain that does not respond to the administration of ibuprofen alone in the first instance	8	*Confirmed consensus at the final meeting*
10	--In case of inefficacy of ibuprofen alone, would you use the fixed combination?	Option Yes *(validated at the first round)*	8	*Confirmed consensus at the final meeting*
11	Derived from the open field of first round In case of inefficacy f ibuprofen alone, would you use the fixed combination?	Headaches	7	*Agreed consensus to all options at the last meeting*^a^
		Chronic rheumatic pain^b^	5	
		Post-traumatic musculoskeletal pain	8	
		Odontalgia	8	
		Moderate pain that does not respond to the administration of paracetamol alone in the first instance	7	*Confirmed consensus at the final meeting*
12	--Would you use the fixed combination for acute pain treatment?^c^	Moderate (4–6 score)	7	*Confirmed consensus at the final meeting*

^a^For questions 9 and 11, independently the minimal difference between the Clinician' answers, the Board agreed that the combination of paracetamol and ibuprofen would be the better choice in case of inefficacy of the 2 drugs as monotherapy especially in case of headache, earache, odontalgia, and musculoskeletal pain

^b^The chronic rheumatic pain was included in the more general group of musculoskeletal pain

^c^Reference was made to the scales approved and used in Italy depending on age: FLACC (Face-Legs-Activity-Crying-Consolability) for children < 3 years, WONG-BAKER for children up to 8 years, NRS (Numeric Rating Scale) for older children

A summary of the main final statements agreed by the Board is reported below. The full results with the clinicians’responses to the each question of second questionnaire are provided in Table 3.

- The Board, almost unanimously, has reached a consensus on the use the fixed combination for the treatment of postoperative pain when both anti-inflammatory and analgesic effects are desired or when paracetamol alone is not sufficient to control a postoperative pain.
- The use suitability of the fixed-dose combination of almost all the post-operative setting with the exclusion of Ear, Nose and Throat (ENT), and abdominal surgery was confirmed, following brainstorming at the final meeting.
- The oral formulation (suspension) of the fixed combination of paracetamol and ibuprofen was judged suitable in pediatrics being able to optimize the dosage in relation to the weight of the child, reduce parental dosing errors as compared to the combined administration of two separate drugs, and allow for quick pain control with greater effectiveness.
- The complementarity of the mechanisms of action, as well as the synergy between the effects of the two substances, were deemed as important factors for the efficacy of the fixed combination by the majority of the Board.
- Greater analgesic power was considered to be the pharmacodynamic advantage of the fixed combination.
- The fixed combination of paracetamol and ibuprofen was deemed suitable when both drugs used in monotherapy are not efficacy, especially for headache, earache, odontalgia, and musculoskeletal pain.
- The majority of our Board members reported their willingness to use the fixed combination of paracetamol and ibuprofen for acute moderate (score 4–6) pain.

## Discussion

The results of this NGT survey highlighted some key points regarding the use of the combination of paracetamol and ibuprofen in the 3.3:1 dose ratio in pediatric age.

The Board agreed that the fixed-dose combination of paracetamol and ibuprofen could be used as a first-choice treatment in moderate (score 4–6) pain in children (assessment pain with Face, Legs, Activity, Cry, Consolability (FLACC) Scale, WONG-BAKER Scale, Numerical Rating Scale (NRS)).

In agreement with the literature [7], the Board agreed on the greater analgesic potency as the main pharmacodynamic advantage of the fixed-dose combination of ibuprofen and paracetamol. The analgesic efficacy of ibuprofen and paracetamol is well known also when used in monotherapy [9]. The superior analgesic effect of the combination of paracetamol and ibuprofen could be ascribed to an enhancement of the efficacy of the single drugs, the greater inhibitory effect on cyclooxygenase (COX) the inhibition of the inflammatory component by ibuprofen, and the activation of the various analgesic mechanisms by paracetamol [12].

The Board was favorable to switching to the fixed combination when paracetamol or ibuprofen in monotherapy is ineffective in treating mild-to-moderate pain, especially in case of headache, earache, odontalgia, and musculoskeletal pain.

In fact, clinical studies in adults on fixed-dose combination of paracetamol and ibuprofen have shown greater efficacy in pain control with better and lasting analgesic effect, and faster onset of action without compromising tolerability, compared to monotherapy [8, 13]. Moreover, the fixed-dose combination of ibuprofen and paracetamol was found to be significantly more effective in preventing persistent pain in pediatrics [14].

The Board reached a consensus on the use of the fixed combination of ibuprofen and paracetamol in the treatment of postoperative pain. ENT surgery, with specific reference to tonsillectomy, was left out from this indication, due to the risk of Post-Tonsillectomy Hemorrhage (PTH) which is a potentially life-threatening complication. Nonetheless, a recent systematic review of the literature and a meta-analysis reported that when ibuprofen is prescribed at the low or high range of commonly used clinical dosages no statistically significant increased risk of PTH [15].

The Board speculate that the low dose of ibuprofen contained in the combination could result in a safer drug profile that could warrant the use in ENT surgeries. On the other hand, further studies are needed to determine if there is a clinically relevant dose-dependent difference in PTH with ibuprofen [15].

The Board did not agree on the use of the paracetamol and ibuprofen combination in postoperative abdominal surgery, as its superior analgesic effect could hide the causes of the pain. Finally, the combined administration of the two drugs, according to the Board, allows for an appropriate dosage based on body weight, thus achieving greater efficacy, and reducing potential dosing errors both on the part of parents and emergency department [5].

## Conclusions

The results of our NGT suggest that the use of the fixed-dose combination of paracetamol and ibuprofen may be a useful option for mild to moderate acute pain control in pediatrics. However, the limited evidence available highlights the need for future well designed studies to better define the advantages of this formulation in the various therapeutic areas.

## Supplementary Information

**Additional file 1. **Search results. 

## Data Availability

Not applicable.

## Abbreviations

IASP: International Association for the Study of Pain
NGT: Nominal Group Technique
ENT: Ear, Nose and Throat
FLACC: Face, Legs, Acfivity, Cry, Consolability
NRS: Numerical Rating Scale
COX: Cyclooxygenase
PTH: Post-Tonsillectomy Hemorrhage
