# Supplementary material for: Paracetamol and ibuprofen combination for the management of acute mild-to-moderate pain in children: expert consensus using the Nominal Group Technique (NGT)
Source: Ital J Pediatr. 2023 Mar 21;49:36. doi: 10.1186/s13052-023-01445-4 (PMC10031994; doi:10.1186/s13052-023-01445-4)
Supplement: Supplementary file 1 — Additional file 1. Search results. [file 13052_2023_1445_MOESM1_ESM.rtf]

Additional file 1. Search results

Terms and connectors search	Results	
Children AND acute pain AND management NOT fever	749	
Children AND pain management AND Therapeutic appropriateness	194	
Paracetamol and ibuprofen combination AND children AND acute pain	10	
Paracetamol and ibuprofen combination AND postoperative pain AND children	15	
Paracetamol and ibuprofen combination AND headache AND children	4	
Paracetamol and ibuprofen combination AND musculoskeletal pain AND children	3	
Paracetamol and ibuprofen combination AND rheumatic pain AND children	4	
Paracetamol and ibuprofen combination AND earache AND children	1	
Paracetamol and ibuprofen combination AND dental pain AND children	3	
